# Modernising continuity: a new conceptual framework

**DOI:** 10.3399/bjgp23X732897

**Published:** 2023-05-26

**Authors:** Emma Ladds, Trish Greenhalgh

**Affiliations:** Nuffield Department of Primary Care Health Sciences, University of Oxford, Oxford.; Nuffield Department of Primary Care Health Sciences, University of Oxford, Oxford.

## INTRODUCTION

Continuity, a cornerstone of general practice, is not only inherently therapeutic^1^ but also a valuable contributor to positive health and wellbeing outcomes. Consistent associations are seen between continuity and higher patient and clinician satisfaction, better measures of health status, and lower mortality.^2^

The long-term, one-to-one relationship between a GP and patient has been in decline for decades, to the regret of many GPs.^3^ Multiple system-level pressures — including rising workload, under-resourcing and staffing, increased policy emphasis on rapid access and plurality of provision, and societal fragmentation resulting in high turnover of patients and staff (especially in deprived localities) — have contributed to a de-prioritisation of continuity within UK general practice. The emergence of multidisciplinary teams covering a wide range of roles^4^ and the introduction and expansion of digital and remote modalities^5^ have created both challenges and opportunities for the delivery of continuity.^6^

## A NEW FRAMEWORK

Academic studies of continuity in primary care have tended to follow a conceptual framework introduced in 2003 by Haggerty *et al*, which distinguished between relational continuity (an ongoing interpersonal relationship between a patient and one or more practitioners), informational continuity (the use of past, recorded information to make current care appropriate for each individual), and managerial continuity (a consistent and coherent approach to the management of health or illness, often disseminated between practitioners and providers).^7^

While Haggerty *et al*’s taxonomy has served us well for 20 years, and several definitions and quantitative metrics are derived from it,^8^ it was not designed for a general practice delivered increasingly through disseminated, remote approaches and modalities. In particular, ‘informational’ and ‘managerial’ continuity, while conceptually distinct, blur into one another in practice, since we use information to manage, document, and transfer care.

We propose a new framework with four elements based on the essence of what is being ‘continued’: the therapeutic relationship; the disease episode; distributed work; and commitment to the practice community. These are summarised in Table 1.

**Box 1.** A new taxonomy of continuity in primary care

**Type of continuity**	**Definition**	**Paradigm**	**Example**
Therapeutic	The patient has an ongoing therapeutic relationship with one or more practitioners, characterised by attentiveness, trust, and positive regard.^1^	Psychodynamic	A patient with medically unexplained symptoms sees her regular GP over many years. Through their dialogue, they work through a complex illness narrative and achieve a degree of ‘containment’ in which she is spared unnecessary investigations, fruitless specialist referrals, and repeated cycles of ‘treatment trials’.
Disease episode	The practitioner who sees a patient for a particular disease episode follows them up until closure OR there is a central integrator (likely the GP but may be a care coordinator) who can interpret/’manage’ the parallel continuities of different practitioners or teams involved in the same disease episode.	Biomedical	A GP trainee receives an online access form from a patient with altered bowel habit. She sends an urgent electronic task through his medical record to the reception team to arrange a face- to- face appointment in which she examines him, orders faecal immunochemical testing and routine blood tests, sets an electronic prompt to check the results when available, telephones the patient to explain there are abnormal findings, arranges a referral under the 2-week wait, and sends the patient a text message link to an online information webpage about the referral process and booking information.
Distributed work	The ‘arc of work’ (the totality of tasks undertaken by a multidisciplinary team of clinical and non-clinical professionals, the patient, and their family or carers, often separated in space and time, and by artificial care distinctions, for example, primary/secondary care boundaries) in order to generate a coordinated experience of coherent care for the patient.^9^	Sociotechnical	An older patient with high blood pressure, heart failure, type 2 diabetes, and asthma is seen regularly by the long-term GP practice nurses who manage chronic disease. She also sees her GP (to fine tune her cardiac medications), GP practice clinical pharmacist (for medication reviews), the community-based heart failure team (at times when her control is poor), and a respiratory consultant (as she later develops chronic obstructive pulmonary disease alongside her asthma). Through details embedded in the electronic record, interfacing electronic systems/platforms, referral and clinical correspondence, and multidisciplinary team or interpersonal discussions, all can ascertain what other team members have been doing, when, and what further input is required. The individual patient and their family or carers are supported to provide as much self-management as possible and are frequently involved in coordinating appointments and communicating as appropriate between the relevant teams.
Commitment to the practice community	A general practice serves a community over time, ensuring access and care according to need, and adapting to changing demographics, cultural values, and policy levers.^10^	Health systems and political economy	A GP practice has served a ‘deep end’ inner-city practice for 60 years. There are high levels of substance abuse, homelessness, and other social problems. Few patients have smart phones. The practice has a turn-up-and-wait policy rather than booked appointments and employs care navigators to support patients with limited system knowledge. They have resisted introducing remote and digital access options because they feel that needy and vulnerable patients would be disadvantaged.

## CONTINUITY IS KEY

Our ongoing ethnographic research in 11 general practices across the UK^5^ suggests that achieving continuity, especially for the most disadvantaged and vulnerable, takes additional resource and creative action from both clinicians and support staff (Ladds E, Greenhalgh T, unpublished data, 2023). It may be tempting to conclude that general practice has become so complex and is under so much pressure that continuity has simply become too difficult to achieve. But these very complexities and pressures mean that it is more important than ever to put effort and resource into delivering continuity in the four key areas of relationships, disease episodes, distributed work, and the communities we serve.

Continuity of relationships is more important than ever, not just because, as the example of medically unexplained symptoms in Table 1 shows, costs and harm can be reduced, but also because, in these stressful times, continuity helps both parties achieve narrative coherence — defined by Merriam-Webster as *‘a systematic logical connection or consistency’* in how experiences are interpreted and ascribed meaning. Therefore, higher continuity makes it easier for both patient and practitioner to maintain a coherent sense of self and has been associated with greater psychological wellbeing; conversely, lower continuity is associated with incoherence, which, in a health system context, may contribute to patient harm or dissatisfaction, low morale or moral injury,^11^ poor retention of staff, and erosion of resilience at health system level.

Continuity of disease episodes is more important than ever because research and analyses of recent significant events have attributed avoidable events (such as death from sepsis, delayed cancer diagnoses, poor palliative care experiences, and safeguarding errors) partly or wholly to breaches in such continuity, with remote and digital modalities portrayed as contributing to these failures.^12^ Moreover, with increasing individuals/services necessarily involved in different disease episodes, potentially each with parallel, internal continuities, the role of an ‘integrator’, for example, the GP or care coordinator, becomes increasingly important. Absent this continuity, the example in Table 1 — an unremarkable patient with altered bowel habit whose stage 1 colon cancer will be promptly diagnosed and easily removed — could ‘slip through the cracks’ and not return until the cancer has metastasised. We must not let such tragedies become our standard of care.

Continuity of distributed work is more important than ever because the less continuity there is in what Strauss called the *‘arc of work’*,^9^ the less efficient and the more stressful that work becomes. As the example of multimorbidity in Table 1 illustrates, continuity of distributed work can make everyone’s job easier and engender a sense of teamwork — both for those around and including the patient. But breaches in this kind of continuity can be particularly draining. At the primary–secondary care interface, for example, clinicians on both sides experience frustrations, delays, and additional work when referral or discharge letters provide inadequate information, when a crucial test result is inaccessible on someone else’s electronic record system, when work perceived to lie within another’s domain is unilaterally transferred, or when patients feel a particularly heavy coordinating or management burden. GPs may feel demoralised and deprofessionalised at what they see as ‘task shifting’. Hospital doctors may feel equally deprofessionalised when their specialist care plans are ignored or implemented half- heartedly, and patients or their families feel forgotten. We must do better.

Continuity of commitment to the practice community is more important than ever because achieving continuity is both more difficult and more crucial for patients from socioeconomically deprived and ethnically diverse groups.^13^ We have known for decades that universal access to basic primary care saves lives.^10^^,^^14^ Evidence is accumulating that remote and digital forms of access compound the ‘inverse care law’ (that people most in need of health care are least likely to seek it or receive it).^15^

Effective solutions to structural disadvantage will not come in the form of individually targeted fixes. As in the example in Table 1 of a ‘deep end’ practice that still has walk-up appointments, solutions may appear paradoxical and take the form of a values-driven push-back against a remote- by-default policy that is judged harmful to the community served.

## CONCLUSION

Rather than gaze wistfully back at an assumed golden age where continuity was defined purely in terms of the one-to-one clinical relationship and was mostly achieved without much difficulty, we need to embrace an extended, multidimensional definition of the term. Understanding the different kinds of continuity shown in Table 1 and exploring the interactions between them, for example, whether their attainment should be viewed independently or as a complementary package, and how their success can be evaluated (both quantitatively and qualitatively) may be a pragmatic and positive move towards regaining some of the meaningful value of continuity.

## References

[1] Balint M (1957). The doctor, his patient, and the illness.

[2] Pereira Gray DJ, Sidaway-Lee K, White E (2018). Continuity of care with doctors—a matter of life and death? A systematic review of continuity of care and mortality. BMJ Open.

[3] Tammes P, Morris RW, Murphy M, Salisbury C (2021). Is continuity of primary care declining in England? Practice-level longitudinal study from 2012 to 2017. Br J Gen Pract.

[4] Gibson J, Francetic I, Spooner S (2022). Primary care workforce composition and population, professional, and system outcomes: a retrospective cross-sectional analysis. Br J Gen Pract.

[5] Greenhalgh T, Shaw SE, Alvarez Nishio A (2022). Remote care in UK general practice: baseline data on 11 case studies. NIHR Open Res.

[6] Ladds E, Khan M, Moore L (2023). The impact of remote care approaches on continuity in primary care: a mixed-studies systematic review. Br J Gen Pract.

[7] Haggerty JL, Reid RJ, Freeman GK (2003). Continuity of care: a multidisciplinary review. BMJ.

[8] Schwarz D, Hirschhorn LR, Kim J-H (2019). Continuity in primary care: a critical but neglected component for achieving high-quality universal health coverage. BMJ Glob Health.

[9] Strauss A (1985). Work and the division of labor. Sociol Q.

[10] World Health Organization (WHO) (2008). Primary health care: now more than ever.

[11] Gibbons SW, Shafer M, Hickling EJ, Ramsey G (2013). How do deployed health care providers experience moral injury?. Narrat Inq Bioeth.

[12] Wieringa S, Neves AL, Rushforth A (2022). Safety implications of remote assessments for suspected COVID-19: qualitative study in UK primary care. BMJ Qual Saf.

[13] Stafford M, Bécares L, Hayanga B (2023). Continuity of care in diverse ethnic groups: a general practice record study in England. Br J Gen Pract.

[14] Starfield B, Shi L, Macinko J (2005). Contribution of primary care to health systems and health. Milbank Q.

[15] Marmot M (2018). An inverse care law for our time. BMJ.

